# Maximum Heart Rate- and Lactate Threshold-Based Low-Volume High-Intensity Interval Training Prescriptions Provide Similar Health Benefits in Metabolic Syndrome Patients

**DOI:** 10.3390/healthcare11050711

**Published:** 2023-02-28

**Authors:** Dejan Reljic, Fabienne Frenk, Hans Joachim Herrmann, Markus Friedrich Neurath, Yurdagül Zopf

**Affiliations:** 1 Department of Medicine 1, University Hospital Erlangen, Friedrich-Alexander University Erlangen-Nürnberg, 91054 Erlangen, Germany; 2 Hector-Center for Nutrition, Exercise and Sports, Department of Medicine 1, University Hospital Erlangen, Friedrich-Alexander University Erlangen-Nürnberg, 91054 Erlangen, Germany; 3 German Center Immunotherapy (DZI), University Hospital Erlangen, Friedrich-Alexander University Erlangen-Nürnberg, 91054 Erlangen, Germany

**Keywords:** obesity, cardiometabolic health, quality of life, interval training, exercise prescription, cardiorespiratory fitness, maximal oxygen uptake, lactate, heart rate, glycemic control

## Abstract

Exercise is an integral part of metabolic syndrome (MetS) treatment. Recently, low-volume high-intensity interval training (LOW-HIIT) has emerged as a time-efficient approach to improving cardiometabolic health. Intensity prescriptions for LOW-HIIT are typically based on maximum heart rate (HR_max_) percentages. However, HR_max_ determination requires maximal effort during exercise testing, which may not always be feasible/safe for MetS patients. This trial compared the effects of a 12-week LOW-HIIT program based on: (a) HR_max_ (HIIT-HR), or (b) submaximal lactate threshold (HIIT-LT), on cardiometabolic health and quality of life (QoL) in MetS patients. Seventy-five patients were randomized to HIIT-HR (5 × 1 min at 80–95% HR_max_), HIIT-LT (5 × 1 min at 95–105% LT) groups, both performed twice weekly on cycle ergometers, or a control group (CON). All patients received nutritional weight loss consultation. All groups reduced their body weight (HIIT-HR: −3.9 kg, *p* < 0.001; HTT-LT: −5.6 kg, *p* < 0.001; CON: −2.6 kg, *p* = 0.003). The HIIT-HR and HIIT-LT groups similarly, improved their maximal oxygen uptake (+3.6 and +3.7 mL/kg/min, *p* < 0.001), glycohemoglobin (−0.2%, *p* = 0.005, and −0.3%, *p* < 0.001), homeostasis model assessment index (−1.3 units, *p* = 0.005, and −1.0 units, *p* = 0.014), MetS z-score (−1.9 and −2.5 units, *p* < 0.001) and QoL (+10 points, *p* = 0.029, and +11 points, *p* = 0.002), while the CON did not experience changes in these variables. We conclude that HIIT-LT is a viable alternative to HIIT-HR for patients who are not able/willing to undergo maximal exercise testing.

## 1. Introduction

The metabolic syndrome (MetS) is a pathology defined by the presence of several cardiometabolic disorders, including obesity (in particular excess abdominal fat), hypertension, dyslipidemia, hyperglycemia and insulin resistance [1]. The occurrence of MetS has significantly risen worldwide during the past decades [2], with the latest estimates suggesting that globally, ~13% to ~31% of adults are affected [3]. Recently, it has been reported that COVID-19 pandemic-related measures like quarantines, social distancing and lockdowns have further contributed to the spread of MetS [4,5]. This trend is alarming because MetS is associated with an increased risk of several serious secondary diseases, such as cardiovascular disease [6], different cancers [7], all-cause mortality [6] and diminished quality of life (QoL) [8]. Additionally, recent observations indicate that excess body weight and the existence of cardiometabolic risk factors constitute an increased risk of developing a critical or lethal disease progression following a COVID-19 infection [9,10]. Therefore, effective therapeutic measures for MetS treatment are probably more urgent now than ever before.

Dietary adaptations, particularly a reduction in caloric intake, and an increase in physical activity are cornerstones in obesity and MetS treatment [11]. While caloric intake restriction is of paramount importance to achieve weight loss [12], it has been demonstrated that physical activity independently lowers the risk of developing several chronic health conditions and premature death, regardless of body mass index (BMI) [13]. It has been suggested that the level of cardiorespiratory fitness (CRF), objectified by the determination of maximal oxygen uptake (VO_2max_), is a major outcome for predicting cardiovascular and all-cause mortality, more significant than other well-established health risk factors like obesity, elevated blood pressure or nicotine abuse [14]. Despite a plethora of evidence on the wide range of health benefits associated with regular exercise, a large part of the global population [15], particularly obese individuals [16], do not meet the minimum physical activity guidelines of 75 min of vigorous-intensity or 150 min of moderate-intensity aerobic activity per week [17]. Over the last decade, surveys have consistently shown that time constraints are among the most frequently reported obstacles to regular exercise, both in the general population [18,19] as well as in clinical cohorts [20,21].

Thus, in the past few years, there has been an increasing scientific interest in designing and evaluating less time-consuming exercise approaches for preventing and treating chronic health conditions [22]. In this regard, low-volume high-intensity interval training (LOW-HIIT) has appeared as an innovative exercise modality to elicit comparable or even greater improvements in CRF and cardiometabolic outcomes in comparison to traditional continuous endurance training [23,24,25,26]. By definition, LOW-HIIT is a particular subtype of interval training involving a total duration of ≤10 min of intense interval bouts of ≥80% of maximum heart rate (HR_max_), embedded in an overall exercise session of ≤30 min (when initial warm-up, recovery between intervals, and cool-down are added up) [23,24]. Recent research from our laboratory [27,28,29,30] and other researchers [31] has demonstrated that LOW-HIIT can effectively improve several cardiometabolic risk factors as well as subjective measures, such as QoL, in obese MetS patients.

Prescriptions for physical exercise are typically based on four main components: frequency, intensity, time, and type of exercise, also referred to as the FIIT principle [32]. Among these, intensity is considered the most important element of the physiological responses to exercise [32]. As with other cardiovascular training types, exercise intensity for LOW-HIIT is most commonly prescribed based on percentages of HR_max_. For a rough estimation of exercise intensity, HR_max_ can be calculated using different formulas [33], most frequently via the “220—age” equation [34]. However, due to high interindividual variability in heart rate (HR) values [34,35], it is rather recommended to directly measure HR_max_ during an exhaustive exercise test in order to obtain more precise results. Although our own studies [27,28,29,30] and data from other research groups [31,36,37] indicate that guideline-based cardiopulmonary exercise testing (CPET) [38] is generally safe and tolerable in clinical settings, maximal exhaustion may be contraindicated in certain patient populations. Furthermore, in some individuals, true HR_max_ may not reached due to peripheral muscular fatigue or a lack of motivation.

Alternatively, exercise intensity can be prescribed using specific physiological thresholds based on ventilatory or blood lactate responses during incremental exercise. Ventilatory (VT) and lactate thresholds (LT) reflect specific submaximal metabolic inflection points of respiratory variables and blood lactate concentration [39]. Ventilatory thresholds and LT have been traditionally used to design training programs and predict performance in endurance and team sports athletes [40,41,42], but threshold-based exercise intensity prescription is also an interesting approach in clinical settings because it does not require maximal effort, making it potentially safer for high-risk patients. Additionally, it has been reported that threshold-based intensity prescriptions for traditional endurance training regimens were superior in improving VO_2max_ in sedentary healthy individuals [43] and cardiometabolic risk factors in MetS patients [44], when compared to intensity prescriptions based on percentages of HR_max_. In this context, endurance training programs involving exercise intensities at or above the LT were found to be particularly effective in lowering blood pressure in type 2 diabetic patients [45] and visceral fat in obese women with MetS [46]. Furthermore, it has been suggested that threshold-based training seems to be related with a lower instance of non-responders to exercise compared to training programs based on maximum values [47]. However, to our knowledge, no research has yet been undertaken to compare the effects of a LOW-HIIT program using HR_max_- versus LT-based exercise intensity prescriptions on cardiometabolic health status in a clinical setting.

Thus, the main objective of this investigation was to compare the effects of a 12-week LOW-HIIT intervention either prescribed based on: (a) percentages of HR_max_ (HIIT-HR) or, (b) LT (HIIT-LT), on various cardiometabolic health indices and QoL in a cohort of obese patients diagnosed with MetS. We hypothesized that both LOW-HIIT protocols would improve cardiometabolic health status and QoL compared with a physically inactive control group but, on the basis of previous research using traditional endurance training regimens [43,44,47], we expected HIIT-LT to provide superior improvements than HIIT-HR.

## 2. Materials and Methods

### 2.1. Study Design

The present investigation was a sub-group analysis of a larger clinical trial examining the impact of various exercise modalities on multiple health outcomes in MetS patients. Other parts of this research project have been previously published elsewhere [27,28,29,30]. The present sub-study presents previously unpublished data from the HIIT-LT group and a sub-sample of the HIIT-HR and a non-exercising control group (CON) that additionally received capillary blood sampling during the CPET for the measurement of lactate concentrations.

In the overall trial, patients were allocated at random to different interval training protocols that were performed 2 times per week for a duration of 12 weeks or to the CON. All patients were provided with standard care nutritional consultation to support their weight reduction. Analogous to the main trial, the key outcome of this investigation was VO_2max_. Further outcomes of interest were cardiometabolic risk indices, body composition and QoL. The sample size determination and randomization process applied in the main trial were reported elsewhere [29]. Briefly, sample size calculation was based on the previous work of Reljic et al. [48], indicating a large effect of LOW-HIIT on VO_2max_ (*d* = 0.97), that resulted in an estimated number of 16 patients per group to yield a statistical power of 95%. To account for dropouts, the aim was to recruit at least 25 patients for randomization into each group. Randomization was preceded by stratification according to VO_2max_, gender, age and BMI using the software MinimPy version 3.0 [49] to reduce the heterogeneity of patients’ main characteristics between groups. The randomization was conducted by a co-worker not engaged in the acquisition and analysis of the data.

All patients were fully informed about the objectives and procedures of the study, which conformed to the Helsinki Declaration, and gave their written consent before being included in the study. The study protocol was approved by the Medical Ethical Committee of the Friedrich-Alexander University Erlangen-Nürnberg (approval number: 210_17B) and registered at ClinicalTrials.gov (ID-number: NCT03306069).

### 2.2. Patients

Patients were recruited via flyers that were posted in medical practices and newspaper advertisements. In a first step, all interested persons were screened for eligibility by phone call or personal visit. The inclusion criteria were: age ≥18 years, a self-reported mainly physically inactive lifestyle as defined previously [50] and clinical diagnosis of MetS as classified by the International Diabetes Federation [51,52]. Criteria for exclusion were: pregnancy, clinical diagnosis of heart disease, oncological diseases, substantial musculoskeletal disorders or other major health limitations that may constitute contraindications to safe participation in exercise. All patients agreed not to change their usual lifestyle habits, apart from the study intervention. Patients were required to attend at least 75% of the scheduled 24 LOW-HIIT sessions to be included in the final analysis.

### 2.3. Health Examinations

One week before starting the intervention, patients received the baseline examination, including several standardized assessments and measurements as described in detail below. The second examination was conducted during the week following the termination of the LOW-HIIT intervention with a minimum 3-day interval between the last training session.

Patients were instructed to appear overnight-fasted, to abstain from alcohol and to avoid strenuous physical activities for at least 24 h prior to each examination. If patients were required to take medication, care was taken to ensure that it was taken at the same time of day for both examinations. Prior to the second examination and regularly during the intervention period, patients were asked whether there had been any changes in the type of medication or dosage taken. The pre- and post-intervention examinations were scheduled at a similar daytime (08:00–08:30 a.m.) to minimize potential circadian bias and lasted approximately 2–3 h for each patient.

All measurements were carried out under stable and standardized laboratory conditions (temperature: 22–24 °C, and humidity: 30–50%) within the examination rooms of the Hector-Center for Nutrition, Exercise and Sports at the University Hospital Erlangen and in the standardized order described below. During the examinations, the patients were dressed in their casual clothes, with an exception for the anthropometric measurements, which were performed in underwear without shoes, and the CPET, for which the patients wore a sport dress or comparable clothing. All examinations were performed by a team of highly experienced personnel, consisting of two study nurses (>5 years of work experience) and an exercise physiologist and physician (>10 years of work experience) who were assisted by two medical students. All the staff involved in the data collection were blinded to the assignment of the patient’s group.

#### 2.3.1. Hydration Testing

After arriving at the research center, patients were first asked to provide a urine sample for a routine screening for urinary tract infections, kidney disorders and diabetes, and for measuring urine specific gravity (USG). Urine sample analyses were conducted within 30 min of collection using Multistix^®^ 10 SG dipsticks (Siemens HealthCare, Erlangen, Germany).

#### 2.3.2. Determination of Blood Pressure and Resting Heart Rate

Following urine collection, patients entered a quiet experimental room and after 5 min rest, resting HR (HR_rest_) and blood pressure were recorded with an automatic upper arm blood pressure monitor (M5 professional, Omron, Mannheim, Germany) [53]. According to recent guidelines [54], systolic (SBP) and diastolic (DBP) blood pressure were measured twice at both upper arms at intervals of 60 s and the average value from the side with the higher blood pressure was recorded. In addition, mean arterial blood pressure (MAB) was estimated according to the following formula [55]:

MAB = DBP + (1/3 [SBP − DBP]).

#### 2.3.3. Blood Collection

After blood pressure measurements, patients remained in the sitting position and venous blood samples were taken from the antecubital area. The blood collection tubes (Sarstedt, Nürmbrecht, Germany) were immediately further prepared and forwarded to the central laboratory of the University Hospital Erlangen for measurement of the serum concentrations of glucose, triglycerides, total cholesterol, low-density (LDL) and high-density lipoprotein cholesterol (HDL) using a photometrical determination method (Clinical Chemistry Analyzer AU700 or AU5800, Beckman Coulter, Brea, CA, USA), glycated hemoglobin A_1c_ (HbA_1c_) using turbidimetric immunoassays (COBAS Integra 400, Roche Diagnostics, Mannheim, Germany) and insulin using a chemiluminescence assay (Liaison XL, DiaSorin, Saluggia, Italy). The homeostasis model assessment index (HOMA-index) was calculated according to the following formula [56]:

Homeostasis model assessment-index = (insulin × glucose)/405.

#### 2.3.4. Anthropometric Measurements

For standardization reasons, patients were asked again to empty their bladder, if necessary, before the measurements. Anthropometric evaluation included measurement of body weight and determination of body composition. More specifically, body weight, fat mass (FM), body fat percentage (FM%), fat free mass (FFM) and total body water (TBW) were determined using a multi-frequency segmental bioelectrical impedance analysis device (seca mBCA 515, Seca, Hamburg, Germany) with confirmed validity [57]. Patients’ waist circumference was measured in the upright position with a flexible tape (Seca, Hamburg, Germany) to the nearest millimeter, at the approximate midpoint between the last touchable rib and the upper iliac crest, as previously described [50].

#### 2.3.5. Determination of the Metabolic Syndrome Severity Score

Metabolic syndrome severity was assessed according to the MetS z-score. The score was calculated using sex-specific equations based on HDL, triglycerides, glucose, waist circumference and MAB, as previously suggested [58]:

Males: [(40 − HDL)/9.0] + [(triglycerides − 150)/81.0] + [(glucose − 100)/11.3] + [(waist circumference−102)/7.7] + [(MAB − 100)/9.1]

Females: [(50 − HDL)/14.1] + [(triglycerides − 150)/81.0] + [(glucose − 100)/11.3] + [(waist circumference − 88)/9.0] + [(MAB − 100)/9.1]

#### 2.3.6. Cardiopulmonary Exercise Testing

Cardiopulmonary exercise testing was performed on a stationary electronically braked cycle ergometer (Corival cpet, Lode, Groningen, The Netherlands) using two different standard exercise protocols [59,60]. Both protocols commenced with a brief familiarization period, followed by measurements from the resting 12-lead electrocardiogram (ECG, custo cardio 110, custo med, Ottobrunn, Germany), blood pressure and respiratory variables. Subsequently, the HIIT-HR and the CON performed a continuously incrementing ramp protocol, beginning at a workload of 50 W and then increasing by 1 W every 5 s (females) and 1 W every 4 s (males), respectively. Using this approach, maximal exertion was typically achieved within 8–12 min, as generally recommended for ramp protocols [59,60]. The HIIT-LT group performed a step incremental test, with a starting workload of 50 W, followed by a stepwise increase in the load by 25 W (females) and 30 W (males), respectively, every 3 min, as recommended to quantify the LT in untrained individuals [60,61]. With the step incremental test, maximum exertion was typically achieved within 10–14 min. Both protocols were performed with a constant cadence ranging between 60–80 rpm until volitional exhaustion.

During all CPET, exercise ECG was permanently monitored (custo cardio 110, custo med, Ottobrunn, Germany) and blood pressure was measured every 2 min with a standard cuff sphygmomanometer (ERKA, Bad Tölz, Germany). An open-circuit breath-by-breath spiroergometric system (Metalyzer 3B-R3, Cortex Biophysik, Leipzig, Germany) was used to continuously measure oxygen uptake (VO_2_) and carbon dioxide output (VCO_2_). At rest, immediately after termination of the exercise and at the 1st, 3rd and 5th min of recovery, 20 µL of capillary blood was sampled from the hyperemized earlobe to measure blood lactate concentrations. In the step incremental test (HIIT-LT group), capillary blood samples were additionally drawn within the last 20 s of each workload stage in order to determine the LT. Blood samples were immediately placed in collection tubes containing a hemolyzing solution, and subsequently measured in our laboratory using an enzymatic-amperometric method (LabTrend, BST Bio Sensor Technology, Berlin, Germany). Upon termination of the exercise, perceived exertion was requested from each patient using the 6–20 Borg scale [62]. Patients had to fulfill a minimum of two of the following criteria [63] in order to assume that maximum exertion had been achieved: a plateau in VO_2_, reaching a peak respiratory exchange ratio (RER_max_) of ≥1.1, a peak blood lactate level of ≥8.0 mmol/L, an age predicted HR_max_ of ≥90% (according to the equation: 220—age) [34] and a perceived exertion value of ≥19 on the Borg scale [62].

#### 2.3.7. Determination of Lactate and Ventilatory Thresholds

The lactate threshold was defined at the workload when blood lactate concentration had reached ≥4 mmol/L, as first established by Mader et al. [64] and later justified by Heck et al. [65]. Since then, the 4 mmol/L LT is also widely referred to as the “onset of lactate accumulation” (OBLA), and broadly used in exercise physiology and practice for performance diagnostics and training prescription [66]. Although it is clear that lactate accumulation does not occur suddenly at a sharp point but rather continuously in a transition zone [67], it is well accepted that the LT frequently corresponds with a blood lactate level of ~4 mmol/L in untrained individuals [68]. Moreover, the fixed 4 mmol/L LT was found to have high reproducibility and predictability in cycling endurance performance [69] and to be useful in prescribing exercise intensity for MetS patients [70]. Determination of HR (HR_LT_) and workload (W_LT_) at the 4 mmol/L LT was performed by applying the software Winlactat version 5.5.2.9 (Mesics, Münster, Germany). First (VT1) and second (VT2, also termed the respiratory compensation point, RCP) ventilatory thresholds were determined independently through visual inspection by two investigators from plots of VCO_2_ and VO_2_ (the V-slope method) [59]. In case of any discrepancy, a consensus was achieved by discussion. Heart rate and workload (W_VT1_ and W_VT2_) at both VTs were identified using an automated software (MetaSoft Studio, Cortex Biophysik, Leipzig, Germany).

### 2.4. Assessment of Self-Reported Quality of Life

Self-reported QoL was measured with the validated EuroQol Group questionnaire (EQ-5D-5L) [71]. The questionnaire consists of the simple EQ visual analogue scale (VAS) ranging from 0–100 (higher ratings imply better QoL) and the EQ-5D index, composed of 5 sub-categories (mobility, self-care, usual activities, pain/discomfort, anxiety/depression, each categorized into 5 severity levels). The values of the 5 sub-categories are transformed into a single variable, with a score of 1.0 representing perfect subjective health and a score of 0 representing the poorest possible health status, respectively [71]. The questionnaires were completed by the patients in a separate waiting lounge at the Hector-Center for Nutrition, Exercise and Sports. Any questions or uncertainties about the questionnaire could be resolved immediately with the investigators.

### 2.5. Monitoring of Daily Nutrition and Nutritional Counseling

Before study enrolment and during the final intervention week, patients were instructed to track their daily food intake over a duration of 3 successive days before each of the two examinations, with the help of a standardized 24 h nutrition protocol (Freiburger Ernährungsprotokoll; Nutri-Science, Freiburg, Germany). After delivery, the protocols were evaluated by a registered dietitian using the software PRODI 6 expert (Nutri-Science, Freiburg, Germany). In addition, patients’ resting metabolic rate (RMR) was estimated using the Harris–Benedict equation [72], as follows:

Males: RMR (kcal/day) = 66.5 + 13.8 × weight (kg) + 5.0 × size (cm]) − 6.8 × age (years)

Females: RMR (kcal/day) = 655 + 9.6 × weight (kg) + 1.8 × size (cm) − 4.7 × age (years)

Based on the food record analysis, anthropometric data and the estimated RMR, patients received individual consultation during a personal conversation with a dietitian to support their weight loss. The dietary recommendations were made in accordance with the current obesity treatment guidelines, targeting a daily calorie reduction of 500 kcal [73]. Furthermore, patients were advised to consume at least 1.0 g/kg of protein per day to counteract a loss of muscle mass during caloric restriction, as previously recommended [74]. After the consultation, patients were provided with handouts, including recipes and nutrient lists, to increase adherence and to support them in the home-based implementation of the nutritional recommendations.

### 2.6. LOW-HIIT Protocols

Patients allocated to the exercise groups performed 2 supervised sessions per week of LOW-HIIT on electronically braked cycle ergometers (Corival cpet, Lode, Groningen, The Netherlands) in our exercise center with a minimum of 1 day recovery between sessions for a total of 12 weeks (24 sessions in total). In order to maximize adherence, patients had the option to schedule their sessions individually during the exercise center’s opening hours. The structure of the LOW-HIIT intervention was in accordance with the protocol introduced by Reljic et al. [48].

In brief, the protocol commenced with a short low-intensity warm-up period of 2 min. Subsequently, patients performed 5 vigorous interval bouts of 1 min duration (by accelerating the cadence and/or increasing the ergometer watt load) divided by 1 min recovery phases. After the fifth interval bout, the protocol concluded with a cool-down of 3 min duration at low intensity, corresponding to an accumulated total duration of 14 min/session. In the HIIT-HR group, patients were instructed to reach a minimum exercise intensity of 80–85% HR_max_ during the intervals for the first 4 weeks. The target intensity during intervals was progressively increased as follows: week 5–8: 85–90% HR_max_ and week 9–12: 90–95% HR_max_. In the HIIT-LT group, the initial minimum exercise intensity to be achieved during intervals was set at a HR corresponding to 95–100% of the LT for the first 4 weeks and then elevated to a HR corresponding to 100–105% of the LT (week 5–12). Patients were equipped with a chest strap HR monitor (Acentas, Hörgertshausen, Germany) in every exercise session, allowing them to follow their HR in real-time on a screen. The HR responses were recorded in every session and later analyzed using the software Heart Rate Monitoring Team System (Acentas, Hörgertshausen, Germany). Average power output and energy expenditure were recorded from the cycle ergometer’s digital displays after each session. Certified sports- and physiotherapists monitored every single session to ensure that the imposed level of exercise intensity was reached.

### 2.7. Statistical Analysis

A priori sample size calculation was conducted using the software G*Power (Heinrich-Heine-University Düsseldorf, Düsseldorf, Germany). Data analyses were performed using the software package SPSS version 24.0 software (IBM Corp., Armonk, NY, USA). Initially, data normality was analyzed using the Shapiro–Wilk test. If the data were normally distributed, a 2 × 2 repeated measures ANOVA was conducted to examine the data for both the main effects (group and time) and interaction effects (group × time). In case of significant results, Holm–Sidak post hoc tests for multiple comparisons were performed. Significant main effects of time were followed by separate post hoc paired t-tests for each group. Levene’s test was utilized to check and verify the homogeneity of variance. If no normal distribution of data was present, log or square root transformation was conducted and the respective statistical analyses were performed with the transformed data. If this procedure did not improve the data heterogeneity (i.e., HbA_1c_, serum insulin concentration, HOMA-index and EQ-5D index), non-parametric tests were used for analysis, including the Friedman two-way analysis of variance by ranks, post hoc Dunn’s Bonferroni tests for group comparisons and Wilcoxon’s tests for within-group comparisons. Effect sizes were evaluated using partial eta-squared (*ηp*^2^) for the ANOVAs and Kendall’s coefficient of concordance (*W*) for the Friedman tests, respectively, and rated as small (0.01–0.05), medium (0.06–0.13) and large (≥0.14) for *ηp*^2^, and small (≤0.10), medium (≥0.30), and large (≥0.50) for *W* [75]. For all analyses, the significance level was set at *p* < 0.05. Data are shown as means ± standard deviation (SD) and pre-/post-intervention changes of the outcome values are reported with 95% confidence intervals (95% CI).

## 3. Results

### 3.1. Study Flow

**Figure 1:**
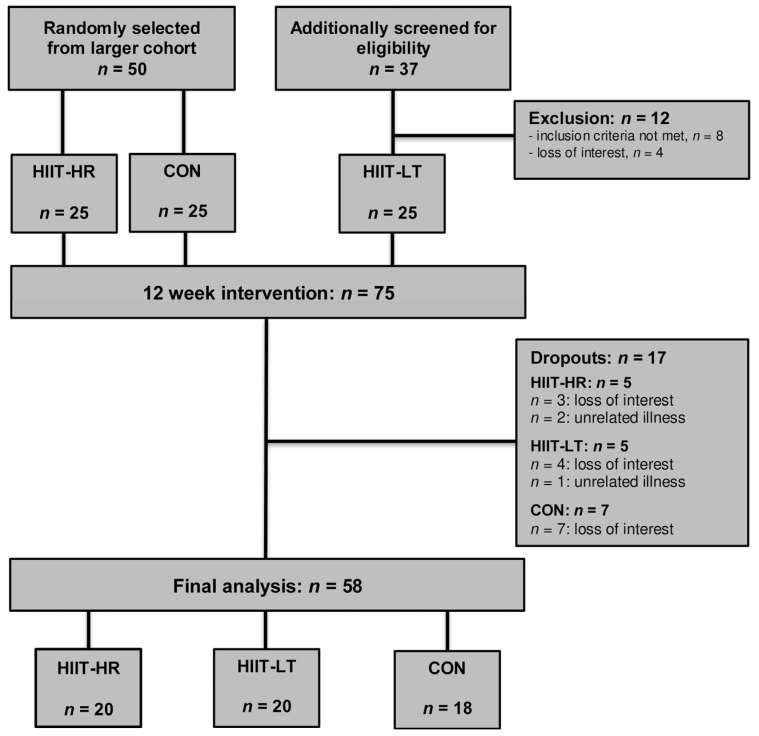
Study flow chart.

Fifty patients (25 each for the HIIT-HR and CON groups) were randomly selected from a larger cohort of the main trial [29] and agreed to additional blood draws during the CPET for the determination of lactate concentrations. Thirty-seven patients were additionally screened for eligibility to be included in the HIIT-LT group until 25 eligible patients were enrolled, resulting in a total sample of 75 patients (25 per group). Seventeen patients dropped out during the study (HIIT-HR = 5, HIIT-LT = 5 and CON = 7). The reasons for dropout are depictured in Figure 1. Consequently, the final analysis involved the data of 58 patients (HIIT-HR = 20, HIIT-LT = 20 and CON = 18). At the baseline, the three groups did not differ significantly in the primary outcome of VO_2max_ and the other main outcomes of interest. Moreover, we did not detect any significant gender effects and therefore, the data of females and males were jointly evaluated in all analyses. Compliance with the LOW-HIIT protocols (the number of scheduled vs. completed exercise sessions) was noticeably high in both exercise groups with 96 ± 6% in the HIIT-HR group and 94 ± 8% in the HIIT-LT group.

### 3.2. Training Data and Adverse Events

The HR_LT_ at the baseline examination corresponded to 94 ± 4% of the HR_max_ in the HIIT-LT group. The average peak HR recorded during each interval bout over the 12 weeks corresponded to 93 ± 7% of the HR_max_ in the HIIT-HR group and 96 ± 3% of the HR_max_ in the HIIT-LT group, respectively, verifying that the target exercise intensity was successfully reached in both groups. The mean session HR (including the warm-up and cool-down phase) corresponded to 79 ± 6% of the HR_max_ in the HIIT-HR group, and 82 ± 5% of the HR_max_ in the HIIT-LT group, respectively. The average peak HR reached during the single intervals and the average session HR were not significantly different between both groups (Table 1). Furthermore, there were no significant differences between both groups in the average power output and energy expenditure per session, with average values of 99.7 ± 24.6 W, 550 ± 149 kilojoules (kJ) and 8.5 ± 2.3 kJ/FFM in the HIIT-HR group, and 105.8 ± 33.2 W, 549 ± 164 kJ and 8.5 ± 2.3 kJ/FFM in the HIIT-LT group, respectively. There were no adverse events observed that were related to the LOW-HIIT.

### 3.3. Hydration Status and Anthropometric Data

During both examinations, the USG values were within the normal ranges for all patients, without significant group differences. There were main effects of time for body weight (*p* < 0.001, *ή*^2^ = 0.54), BMI (*p* < 0.001, *ή*^2^ = 0.54), FM (*p* < 0.001, *ή*^2^ = 0.42), FM% (*p* < 0.001, *ή*^2^ = 0.29), FFM (*p* = 0.003, *ή*^2^ = 0.15), TBW (*p* < 0.001, *ή*^2^ = 0.21) and waist circumference (*p* < 0.001, *ή*^2^ = 0.56). Furthermore, there was a group-by-time interaction for waist circumference (*p* < 0.001, *ή*^2^ = 0.25) and trend toward an interaction effect for FM (*p* = 0.055, *ή*^2^ = 0.10) All groups significantly reduced their body weight (HIIT-HR: −3.9 kg, 95% CI: −5.5 to −2.3 kg, *p* < 0.001; HIIT-LT: −5.6 kg, 95% CI: −7.8 to −3.4 kg, *p* < 0.001; CON: −2.6 kg, 95% CI: −4.2 to −1.0 kg, *p* = 0.003). The quantity of weight loss was not significantly different between the three groups (*p* = 0.064), but compared to the exercise groups, the CON did not significantly reduce FM and waist circumference. Compared to the CON, the decrease in waist circumference was larger in the HIIT-HR (−7 cm, 95% CI: −9 to −1 cm, *p* = 0.010) and HIIT-LT (−8 cm, 95% CI: −11 to −3 cm, *p* < 0.001) groups. Table 2 displays all group specific pre-/post-intervention anthropometric variables.

### 3.4. Nutrition Data

There was a main effect of time for energy (*p* = 0.024, *ή*^2^ = 0.09) and fat intake (*p* = 0.005, *ή*^2^ = 0.14). Post hoc tests indicated that the reduction in energy and fat intake per day only reached statistical significance in the CON (−410 kcal, 95% CI: −747 to −72 kcal, *p* = 0.020, and −30 g, 95% CI: −46 to −8 g, *p* = 0.007, respectively), however, there were no significant differences in daily calorie reduction between the groups. Pre- and post-intervention, there were no significant group differences in nutritional intake (Table 3).

### 3.5. Cardiopulmonary Exercise Testing Data

During both CPET examinations, all patients fulfilled at least two maximal exertion criteria [63]. In all three groups, there were no significant differences in pre- and post-intervention resting lactate concentrations (HIIT-HR: 1.2 ± 0.3 and 1.1 ± 0.2 mmol/L, HIIT-LT: 1.1 ± 0.2 and 1.0 ± 0.3 mmol/L, CON: 1.2 ± 0.3 and 1.2 ± 0.2 mmol/L), maximal lactate levels (HIIT-HR: 7.5 ± 1.7 and 7.7 ± 2.1 mmol/L, HIIT-LT: 7.9 ± 1.5 and 7.9 ± 2.6 mmol/L, CON: 7.9 ± 1.8 and 7.5 ± 1.8 mmol/L) RER_max_ (HIIT-HR: 1.03 ± 0.1 and 1.04 ± 0.1, HIIT-LT: 1.02 ± 0.1 and 1.03 ± 0.1, CON: 1.01 ± 0.1 and 1.01 ± 0.1) and HR_max_ (HIIT-HR: 158 ± 17 and 160 ± 18 b/min, HIIT-LT: 156 ± 21 and 158 ± 19 b/min, CON: 158 ± 21 and 157 ± 21 b/min), indicating that maximal exhaustion levels were similar during both examinations. In the HIIT-LT group, there was a high agreement between W_LT_ and W_VT2_ (*p* < 0.001, *r* = 0.78).

A main effect of time and group-by-time interaction was found for relative (*p* < 0.001, *ή*^2^ = 0.50, and *p* < 0.001, *ή*^2^ = 0.39, respectively) and absolute VO_2max_ (*p* < 0.001, *ή*^2^ = 0.32, and *p* < 0.001, *ή*^2^ = 0.42, respectively), relative (*p* < 0.001, *ή*^2^ = 0.67, and *p* < 0.001, *ή*^2^ = 0.48, respectively) and absolute W_max_ (*p* < 0.001, *ή*^2^ = 0.66, and *p* < 0.001, *ή*^2^ = 0.62, respectively), W_VT1_ (*p* < 0.001, *ή*^2^ = 0.64, and *p* < 0.001, *ή*^2^ = 0.58, respectively) and W_VT2_ (*p* = 0.026, *ή*^2^ = 0.10, and *p* < 0.001, *ή*^2^ = 0.39, respectively). Additionally, there was a main effect of time for HR_VT1_ (*p* = 0.002, *ή*^2^ = 0.21) and HR_VT2_ (*p* < 0.001, *ή*^2^ = 0.26).

The HIIT-HR and HIIT-LT groups showed similar improvements in relative VO_2max_ (3.6 mL/kg/min, 95% CI: 2.5 to 4.7 mL/kg/min, *p* < 0.001, and 3.7 mL/kg/min, 95% CI: 2.3 to 5.0 mL/kg/min, *p* < 0.001), absolute VO_2max_ (301 mL/min, 95% CI: 194 to 409 mL/min, *p* < 0.001, and 257 mL/min, 95% CI: 154 to 360 mL/min, *p* < 0.001), relative W_max_ (0.3 W/kg, 95% CI: 0.2 to 0.4 W/kg, *p* < 0.001, and 0.3 W/kg, 95% CI: 0.2 to 0.4 W/kg, *p* < 0.001), absolute W_max_ (25 W, 95% CI: 20 to 30 W, *p* < 0.001, and 26 W, 95% CI: 20 to 31 W, *p* < 0.001), W_VT1_ (30 W, 95% CI: 24 to 35 W, *p* < 0.001, and 30 W, 95% CI: 22 to 38 W, *p* < 0.001) and W_VT2_ (17 W, 95% CI: 5 to 30 W, *p* = 0.012, and 22 W, 95% CI: 12 to 32 W, *p* < 0.001). In the HIIT-LT group, there was a significant increase in W_LT_ (12 W, 95% CI: 3 to 22 W, *p* = 0.012). None of these outcomes improved in the CON. By contrast, absolute VO_2max_ (−214 mL/min, 95% CI: −221 to −8 mL/min, *p* = 0.037) and W_VT_ (−17 W, 95% CI: −28 to −7 W, *p* = 0.003) decreased from pre- to post-intervention.

Compared to the CON, the HIIT-HR and HIIT-LT groups exhibited significantly greater increases in relative VO_2max_ (4.0 mL/kg/min, 95% CI: 2.0 to 5.9 mL/kg/min, *p* < 0.001, and 4.1 mL/kg/min, 95% CI: 2.1 to 5.9 mL/kg/min, *p* < 0.001), absolute VO_2max_ (415 mL/min, 95% CI: 237 to 594 mL/min, *p* < 0.001, and 371 mL/min, 95% CI: 196 to 547 mL/min, *p* < 0.001), relative W_max_ (0.3 W/kg, 95% CI: 0.2 to 0.4 W/kg, *p* < 0.001, and 0.3 W/kg, 95% CI: 0.2 to 0.4 W/kg, *p* < 0.001), absolute W_max_ (30 W, 95% CI: 21 to 38 W, *p* < 0.001, and 30 W, 95% CI: 21 to 39 W, *p* < 0.001), W_VT1_ (32 W, 95% CI: 12 to 53 W, *p* < 0.001, and 40 W, 95% CI: 19 to 60 W, *p* < 0.001) and W_VT2_ (35 W, 95% CI: 16 to 54 W, *p* < 0.001, and 40 W, 95% CI: 20 to 59 W, *p* < 0.001). Pre-/post-intervention CPET outcomes for each group are shown in Table 4.

### 3.6. Cardiometabolic Data

A group-by-time interaction was found for SBP (*p* < 0.001, *ή*^2^ = 0.29), DBP (*p* < 0.001, *ή*^2^ = 0.26), MAB (*p* < 0.001, *ή*^2^ = 0.33) and the MetS z-score (*p* < 0.001, *ή*^2^ = 0.30). Additionally, a main effect of time was observed for HR_rest_ (*p* < 0.001, *ή*^2^ = 0.33), SBP (*p* < 0.001, *ή*^2^ = 0.43), DBP (*p* < 0.001, *ή*^2^ = 0.40), MAB (*p* < 0.001, *ή*^2^ = 0.48), HbA_1c_ levels (*p* < 0.001, *W =* 0.23), serum insulin concentration (*p* < 0.001, *W* = 0.25), HOMA-index (*p* < 0.001, *W* = 0.28) and the MetS z-score (*p* < 0.001 *ή*^2^ = 0.61).

Post hoc tests showed that both in the HIIT-HR and HIIT-LT groups, there were significant reductions in HR_rest_ (−6 b/min, 95% CI: −9 to −2 b/min, *p* = 0.006, and −6 b/min, 95% CI: −7 to −3 b/min, *p* < 0.001), SBP (−11 mmHg, 95% CI: −15 to −7 mmHg, *p* < 0.001, and −13 mmHg, 95% CI: −16 to −9 mmHg, *p* < 0.001), DBP (−8 mmHg, 95% CI: −11 to −4 mmHg, *p* < 0.001, and −10 mmHg, 95% CI: −13 to −7 mmHg, *p* < 0.001), MAB (−9 mmHg, 95% CI: −12 to −6 mmHg, *p* < 0.001, and −11 mmHg, 95% CI: −14 to −8 mmHg, *p* < 0.001), HbA_1c_ levels (−0.2%, 95% CI: −0.4 to −0.1%, *p* = 0.012, and −0.3%, 95% CI: −0.4 to −0.2%, *p* < 0.001), serum insulin concentrations (−5 µU/mL, 95% CI: −8 to −1 µU/mL, *p* = 0.007, and −3 µU/mL, 95% CI: −8 to −2 µU/mL, *p* = 0.019), HOMA-index (−1.3 units, 95% CI: −2.4 to −0.2 units, *p* = 0.005, and −1.0 units, 95% CI: −2.1 to −0.2 units, *p* = 0.014) and the MetS z-score (−1.9 units, 95% CI: −2.6 to −1.8 units, *p* < 0.001, and −2.5 units, 95% CI: −3.0 to −2.0 units, *p* < 0.001). No significant changes occurred in the CON.

Compared to the CON, the HIIT-HR and HIIT-LT groups showed significantly greater pre-/post-intervention reductions in SBP (−12 mmHg, 95% CI: −19 to −4 mmHg, *p* < 0.001, and −13 mmHg, 95% CI: −20 to −5 mmHg, *p* < 0.001), DBP (−8 mmHg, 95% CI: −13 to −2 mmHg, *p* = 0.007, and −10 mmHg, 95% CI: −16 to −4 mmHg, *p* < 0.001), MAB (−9 mmHg, 95% CI: −15 to −4 mmHg, *p* < 0.001, and −11 mmHg, 95% CI: −17 to −6 mmHg, *p* < 0.001) and MetS z-score (−1.6 units, 95% CI: −2.6 to −0.5 units, *p* < 0.001, and −2.2 units, 95% CI: −3.2 to −1.1 units, *p* < 0.001). Pre-/post-intervention cardiometabolic variables for each group are shown in Table 5.

### 3.7. Self-Reported Quality of Life Data

A main effect of time was detected for EQ-VAS (*p* < 0.001, *ή*^2^ = 0.24) and the EQ-5D index (*p* = 0.031, *W* = 0.08). Both the HIIT-HR and HIIT-LT groups experienced a pre-/post-intervention increase in EQ-VAS (10 points, 95% CI: 1 to 18 points, *p* = 0.029, and 11 points, 95% CI: 5 to 17 points, *p* = 0.002), whereas no significant changes were recorded in the CON (Table 6).

## 4. Discussion

Exercise intensity is a crucial—if not the most pivotal—variable in exercise prescription [33]. Intensity prescriptions for (LOW-)HIIT programs are typically based on percentages of the HR_max_, which, however, may be associated with several limitations in clinical populations. Given the rising popularity of LOW-HIIT in prevention programs and clinical exercise interventions, it is timely to investigate the viability of alternative approaches for exercise intensity prescriptions in individuals, where determination of the HR_max_ may not be feasible. To our knowledge, this investigation was the first to compare the effects of a LOW-HIIT intervention based on either the HR_max_ or the submaximal LT in obese patients with MetS. The major result was that the HIIT-HR and HIIT-LT produced similar improvements in key cardiometabolic outcomes and self-reported QoL after a period of 12 weeks.

The finding that the two LOW-HIIT protocols had similar beneficial effects on cardiometabolic health and QoL was in contrast to our hypothesis based on some previous research, reporting that threshold-based exercise intensity prescriptions are superior to relative percent concepts in improving various cardiometabolic outcomes [43,44,47]. When analyzing the training data (average HR and power output), however, it becomes evident that the physiological demands were comparable between both LOW-HIIT protocols. Furthermore, compliance with both protocols was similarly very high (HIIT-HR: 96 ± 6%, and HIIT-LT: 94 ± 8%) and thus, it is plausible that both protocols yielded similar benefits. In this context, it is noteworthy that 4 mmol/L LT data for obese MetS patients have rarely been described in the literature. We found that the HR_LT_ corresponded to 94 ± 4% of the HR_max_ in our patients, which is in accordance with the well-established 3-phase model introduced by Skinner et al. [76], illustrating that the HR at the 4 mmol/L LT typically exceeds 90% of the HR_max_.

When comparing both LOW-HIIT protocols, it is notable that all patients in our study were able and willing to reach maximal exertion during the CPET. Thus, in general, if patients are physically able and no symptoms occur during exercise that would require premature termination, we recommend that CPET should be performed until exhaustion in order to acquire maximum performance data and to use the established criteria to verify that maximum exertion has been reached [63]. However, it is an important practical result of this investigation that exercise intensity prescription for the LOW-HIIT protocols can also be feasibly generated using a submaximal exercise test until the LT is reached, which may constitute a viable approach if maximal CPET is contraindicated or patients are not able/motivated to exercise until exertion.

Both LOW-HIIT protocols induced improvements in several health-related outcomes that can be considered clinically meaningful. First, patients involved in the LOW-HIIT improved VO_2max_ by ~3.7 mL/kg/min. The importance of CRF for health and longevity has been well-established in decades of research [13,14,77]. It has been reported, for example, that each VO_2max_ increase by 1 mL/kg/min is associated with a 9% risk decrease in overall mortality [78]. Recent large-scale research verified these findings, demonstrating that each 3.5 mL/kg/min improvement in CRF is related to a decreased risk of premature death due to cardiovascular disease and cancer each by 15% [79]. Second, the reduction in MetS z-score indicates an improvement in overall MetS severity, which was mainly related to reductions in blood pressure (−12 mmHg SBP/−9 mmHg DBP, on average) and waist circumference (−8 cm, on average). Large prospective cohort studies have indicated a reduced risk of coronary heart disease by 22% and stroke by 41%, respectively, per each −10 mmHg SBP/−5 mmHg DBP decrease [80] and an 8% reduction in all-cause mortality per −5 cm decrease in waist circumference [81]. Additionally, both LOW-HIIT protocols had beneficial effects on glucose metabolism as indicated by significant reductions in the HbA_1c_ levels, fasting insulin and HOMA-index. Improvements in these outcomes have been associated with improved cardiometabolic health [82] and a lower risk of colorectal cancer [83], for example. Third, self-reported QoL improved in response to both LOW-HIIT protocols. The mean pre-intervention EQ-VAS scores were markedly lower in our patient cohort than the values reported for the general population [71], which is in line with data from other researchers indicating a relationship between MetS and a diminished QoL [8]. The marked post-intervention improvement in EQ-VAS following LOW-HIIT underscores the well-established association between physical activity [84], CRF levels [85] and enhanced QoL.

Taken together, these findings highlight the pleiotropic effects of exercise on a broad range of important health markers and support the “exercise is medicine” message [86]. Although we clearly recommend that patients who are willing and capable of being more physically active should be encouraged to perform higher volumes of exercise in order maximize the health benefits, our observations provide further evidence [22,23,24,25,26] that even very small doses of targeted exercise can provide meaningful improvements in the physiological and psychological outcomes.

The three groups achieved an average weight loss of ~3.5% within the 12-week study period, which is in accordance with most lifestyle-intervention programs for obesity [87]. It is noteworthy that the relative weight loss amounts tended to be greater in the two exercise groups compared to the CON, but the total difference did not reach statistical significance (exercise groups vs. CON, *p* = 0.066). This finding is not surprising as the three groups did not significantly differ in the amount of caloric reduction, which is the key component to achieve a negative energy balance and to reduce body weight [88]. Although there is evidence that (LOW-)HIIT, compared to traditional continuous endurance training, may have different (more pronounced) effects on some physiological factors associated with weight loss, including higher excess post-exercise oxygen consumption [89], stronger post-exercise suppression of appetite perception [90] or greater changes in concentrations of distinctive gut hormones and leptin [91], our results suggest that the extremely low volume of exercise applied in the present study did not have a substantial impact on the daily overall energy balance. Thus, when it comes to pure weight loss, higher-volume exercise modalities with greater energy expenditure (e.g., longer-lasting endurance exercise or HIIT involving more and/or longer intervals) may be more effective compared to our very low-volume HIIT protocol. However, in agreement with previous reports, it is too short-sighted to define a successful obesity treatment solely in terms of pure weight loss because it is more important to improve the CRF and other cardiometabolic health outcomes than to strictly follow anthropometric measures to improve morbidity and longevity [13,14,77]. In this regard, we observed substantial differences between the patients allocated to the CON and those performing LOW-HIIT, with only the “exercisers” achieving significant improvements in cardiometabolic health and QoL, despite similar weight loss.

Finally, we note some potential limitations to this investigation. Firstly, we note that all patients received standard care nutritional counseling in addition to the LOW-HIIT, which may represent a confounding variable for the observed pre-/post intervention changes. However, we do not feel that the nutritional modification had any meaningful effect on the major research question of this study (HIIT-HR vs. HIIT-LT) because both groups received the same counseling and there were no significant differences in the nutritional intake between the HIIT-HR and HIIT-LT groups. Nevertheless, it cannot be completely ruled out that potential within- or between-group variations in nutrition might have affected the results to some extent.

Secondly, we are well aware that numerous LT as well as VT concepts exist [40,41,60,61,66,67,69] and one can argue why we used the fixed 4 mmol/L LT [64,65] to prescribe the exercise intensity to the HIIT-LT group. Specific reasons for selecting the 4 mmol/L LT are given in the methodology section, but we highlight that it was the major aim of this study to compare the effects of LOW-HIIT prescriptions based on maximal versus submaximal exercise parameters and not to investigate which threshold concept might be the best for obese MetS patients. Nevertheless, we do not rule out that another threshold concept/exercise prescription approach may have achieved even better results or might even have been superior to the HR_max_-based prescription method. Future research may wish to explore this important question. Moreover, further research is necessary to investigate whether the findings obtained by this specific cohort of obese MetS patients may be transferred to other (clinical) populations. Lastly, it must be considered that all the examinations and the LOW-HIIT intervention were carried out in a well-controlled clinical environment. Thus, it remains to be elucidated to which degree our findings can be applied to non-clinical settings.

## 5. Conclusions

The HIIT-HR and HIIT-LT induced similar improvements in cardiometabolic health and QoL in obese MetS patients. Thus, the practical take-home message for clinicians and exercise physiologists who wish to implement LOW-HIIT in clinical populations, is that exercise intensity can feasibly and effectively be prescribed using a submaximal LT-based exercise test if patients are not willing or able to perform maximal CPET.

## Figures and Tables

**Table 1:** Average heart rate during intervals and during the whole exercise session ^1^.

Variable	HIIT-HR	HIIT-LT
Week 1–4		
Whole session (%HR_max_)	79.1 ± 5.7	81.8 ± 4.8
Intervals (%HR_max_)	92.6 ± 6.0	95.5 ± 3.4
Week 5–8		
Whole session (%HR_max_)	78.2 ± 7.7	81.6 ± 5.4
Intervals (%HR_max_)	92.3 ± 7.4	95.4 ± 4.1
Week 9–12		
Whole session (%HR_max_)	78.0 ± 7.3	82.0 ± 6.1
Intervals (%HR_max_)	92.7 ± 8.2	95.6 ± 3.7

Values are presented as mean ± SD. HR_max_ = maximum heart rate. ^1^ including warm up, intervals, recovery periods and cool-down.

**Table 2:** Anthropometric and hydration variables.

Variable	HIIT-HR (*n* = 20)		HIIT-LT (*n* = 20)		CON (*n* = 18)	
	Pre	Post	Pre	Post	Pre	Post
Weight (kg)	117.1 ± 30.3	113.2 ± 29.3 ^c^	117.5 ± 24.5	111.9 ± 24.4 ^c^	110.7 ± 21.8	108.1 ± 23.2 ^b^
BMI (kg/m^2^)	38.0 ± 7.7	36.8 ± 7.5 ^c^	39.5 ± 8.2	37.5 ± 8.2 ^c^	37.3 ± 5.1	36.4 ± 5.7 ^b^
FM (kg)	50.0 ± 17.6	46.3 ± 16.4 ^c^	52.5 ± 17.4	47.8 ± 18.2 ^c^	49.8 ± 11.4	48.3 ± 13.7
FM (%)	42.3 ± 8.2	40.5 ± 8.5 ^c^	44.0 ± 7.0	41.8 ± 7.9 ^b^	45.0 ± 6.2	44.5 ± 7.1
FFM (kg)	67.1 ± 17.0	67.1 ± 18.0	65.1 ± 11.4	64.1 ± 11.1	60.9 ± 14.3	59.8 ± 13.8 ^b^
TBW (L)	50.0 ± 13.0	49.8 ± 13.3	48.7 ± 8.4	47.8 ± 8.1 ^a^	45.9 ± 10.3	44.9 ± 10.0 ^c^
USG (mg/dL)	1025 ± 12	1022 ± 11	1026 ± 14	1023 ± 12	1024 ± 10	1023 ± 10
Waist (cm)	116.8 ± 21.3	110.2 ± 18.1 ^c^	119.4 ± 13.6	111.0 ± 15.0 ^c^	113.3 ± 13.4	111.8 ± 15.4

Values are presented as mean ± SD. BMI = body mass index, FM = fat mass, FFM = skeletal muscle mass, TBW = total body water, USG = urine specific gravity. ^a^ (*p* < 0.05), ^b^ (*p* < 0.01), ^c^ (*p* < 0.001): significantly different compared to pre-intervention.

**Table 3:** Nutritional intake.

Variable	HIIT-HR (*n* = 20)		HIIT-LT (*n* = 20)		CON (*n* = 18)	
	Pre	Post	Pre	Post	Pre	Post
Energy (kcal/d)	2320 ± 669	2111 ± 875	2359 ± 1204	1966 ± 790	2206 ± 606	1796 ± 620 ^a^
Protein (g/d)	96.8 ± 42.4	96.9 ± 37.9	107.3 ± 95.7	97.1 ± 60.9	94.0 ± 26.0	86.2 ± 30.5
Protein (g/kg/d)	0.9 ± 0.3	0.9 ± 0.3	1.0 ± 0.5	0.9 ± 0.6	0.9 ± 0.3	0.9 ± 0.3
Fat (g/d)	95.4 ± 39.4	83.3 ± 39.1	86.7 ± 41.3	76.7 ± 41.2	93.9 ± 32.1	66.6 ± 26.2 ^b^
Fat (g/kg/d)	0.9 ± 0.3	0.8 ± 0.3	0.8 ± 0.3	0.7 ± 0.4	0.9 ± 0.3	0.7 ± 0.3 ^a^
CHO (g/d)	211.3 ± 50.1	220.6 ± 106.5	233.7 ± 104.8	186.7 ± 73.8	212.2 ± 73.0	183.9 ± 83.6
CHO (g/kg/d)	1.9 ± 0.6	2.0 ± 0.7	2.1 ± 1.0	1.7 ± 0.7	2.0 ± 0.8	1.8 ± 0.9
Fiber (g/d)	24.3 ± 9.4	23.7 ± 12.0	21.1 ± 9.0	23.4 ± 11.4	22.8 ± 13.4	22.0 ± 8.8

Values are presented as mean ± SD. CHO = carbohydrates. ^a^ (*p* < 0.05), ^b^ (*p* < 0.01): significantly different compared to pre-intervention.

**Table 4:** Cardiopulmonary exercise testing variables.

Variable	HIIT-HR (*n* = 20)		HIIT-LT (*n* = 20)		CON (*n* = 18)	
	Pre	Post	Pre	Post	Pre	Post
VO_2max_ (mL/kg/min)	21.6 ± 4.8	25.2 ± 5.3 ^c^	22.0 ± 6.9	25.7 ± 7.4 ^c^	21.6 ± 7.0	21.2 ± 7.4
VO_2max_ (L/min)	2.4 ± 0.6	2.7 ± 0.7 ^c^	2.5 ± 0.6	2.8 ± 0.7 ^c^	2.4 ± 0.8	2.2 ± 0.8 ^a^
W_max_ (W/kg)	1.4 ± 0.4	1.7 ± 0.4 ^c^	1.4 ± 0.5	1.7 ± 0.6 ^c^	1.4 ± 0.5	1.4 ± 0.5
W_max_ (W)	156.6 ± 42.0	181.3 ± 38.8 ^c^	156.7 ± 45.1	182.2 ± 49.6 ^c^	153.3 ± 57.7	148.5 ± 53.7
W_VT1_ (W)	58.0 ± 25.3	87.3 ± 24.0 ^c^	63.4 ± 26.2	93.1 ± 30.2 ^c^	62.2 ± 33.8	57.1 ± 28.6
W_VT2_ (W)	125.2 ± 35.8	142.4 ± 33.4 ^a^	132.6 ± 35.3	154.6 ± 38.7 ^c^	136.0 ± 41.6	119.2 ± 41.0 ^b^
W_LT_ (W) *	---	---	138.9 ± 38.8	151.3 ± 46.7 ^a^	---	---

Values are presented as mean ± SD. VO_2max_ = maximal oxygen uptake, W_max_ = maximal power output, W_VT1_, W_VT2_ and W_LT_ = power output at ventilatory threshold 1, power output at ventilatory threshold 2 and power output lactate threshold, respectively. ^a^ (*p* < 0.05), ^b^ (*p* < 0.01), ^c^ (*p* < 0.001): significantly different compared to pre-intervention. * only determined in the HIIT-LT group.

**Table 5:** Cardiometabolic variables.

Variable	HIIT-HR (*n* = 20)		HIIT-LT (*n* = 20)		CON (*n* = 18)	
	Pre	Post	Pre	Post	Pre	Post
HR_rest_ (b/min)	75.7 ± 9.8	70.3 ± 8.0 ^b^	74.6 ± 12.4	69.4 ± 12.3 ^c^	77.3 ± 8.8	74.7 ± 10.0
SBP (mmHg)	143.3 ± 13.1	132.0 ± 11.6 ^c^	140.0 ± 13.6	127.4 ± 13.4 ^c^	137.5 ± 10.9	137.8 ± 7.8
DBP (mmHg)	93.5 ± 7.8	86.0 ± 7.0 ^c^	88.6 ± 9.7	78.4 ± 7.4 ^c^	88.3 ± 8.9	88.4 ± 7.6
MAB (mmHg)	110.1 ± 8.3	101.2 ± 7.2 ^c^	105.7 ± 10.2	94.8 ± 7.8 ^c^	104.8 ± 8.8	105.0 ± 6.4
Glucose (mg/dL)	103.0 ± 18.6	101.0 ± 13.2	104.9 ± 14.1	99.8 ± 15.0	94.1 ± 17.4	92.6 ± 14.3
HbA_1c_ (%)	5.7 ± 0.5	5.5 ± 0.4 ^b^	5.7 ± 0.4	5.4 ± 0.4 ^c^	5.6 ± 0.9	5.6 ± 0.7
Triglycerides (mg/dL)	135.2 ± 59.2	130.0 ± 35.0	116.5 ± 45.1	119.4 ± 55.4	120.0 ± 90.0	119.1 ± 62.4
Cholesterol (mg/dL)	219.3 ± 35.1	215.3 ± 36.2	208.8 ± 36.4	211.5 ± 34.4	218.0 ± 37.2	217.0 ± 31.1
LDL (mg/dL)	147.8 ± 28.8	143.9 ± 26.8	140.7 ± 28.0	141.2 ± 29.3	148.7 ± 29.9	148.2 ± 22.6
HDL (mg/dL)	49.2 ± 10.0	49.4 ± 11.2	47.0 ± 9.4	49.8 ± 12.0	55.1 ± 12.3	53.0 ± 12.1
Insulin (µU/mL)	18.3 ± 11.7	12.7 ± 9.3 ^b^	19.7 ± 10.8	17.3 ± 12.8 ^a^	18.2 ± 12.6	15.9 ± 8.0
HOMA-index	4.8 ± 3.6	3.4 ± 2.1 ^b^	5.2 ± 3.0	4.2 ± 3.0 ^a^	4.4 ± 3.6	3.8 ± 2.4
MetS z-score	3.3 ± 4.0	1.4 ± 3.0 ^c^	3.3 ± 2.6	0.8 ± 3.0 ^c^	2.0 ± 2.8	1.5 ± 3.1

Values are presented as mean ± SD. HR_rest_ = resting heart rate, SBP = systolic blood pressure, DBP = diastolic blood pressure, MAB = mean arterial blood pressure, HbA_1c_ = glycated hemoglobin A_1c_, LDL = low-density lipoprotein cholesterol, HDL = high-density lipoprotein cholesterol, HOMA = homeostasis model assessment, MetS = metabolic syndrome. ^a^ (*p* < 0.05), ^b^ (*p* < 0.01), ^c^ (*p* < 0.001): significantly different compared to pre-intervention.

**Table 6:** Quality of life variables.

Variable	HIIT-HR (*n* = 20)		HIIT-LT (*n* = 20)		CON (*n* = 18)	
	Pre	Post	Pre	Post	Pre	Post
EQ-VAS	63.0 ± 15.3	72.6 ± 21.1 ^a^	63.9 ± 15.7	74.7 ± 17.2 ^b^	60.4 ± 25.1	65.7 ± 28.3
EQ-5D index	0.84 ± 0.16	0.86 ± 0.20	0.85 ± 0.12	0.88 ± 0.14	0.87 ± 0.14	0.86 ± 0.19

Values are presented as mean ± SD. BMI = body mass index, FM = fat mass, FFM = skeletal muscle mass, TBW = total body water. ^a^ (*p* < 0.05), ^b^ (*p* < 0.01): significantly different compared to pre-intervention.

## Data Availability

The datasets generated and analyzed during the current study are not publicly available but are available from the corresponding author on reasonable request.
